# Smilax glabra flavonoids ameliorate non-alcoholic steatohepatitis by regulating PKM2-dependent glycolysis and suppressing AIM2 inflammasome activation

**DOI:** 10.3389/fimmu.2026.1906439

**Published:** 2026-09-09

**Authors:** Ziyao Pang, Yue Ding, Leyi Hu, Shihao Ding, Zhaowei Cai, Dejun Wang

**Affiliations:** 1 Laboratory Animal Research Center, Academy of Chinese Medical Sciences, Zhejiang Chinese Medical University, Hangzhou, China; 2 School of Pharmacy, Zhejiang Chinese Medical University, Hangzhou, China

**Keywords:** AIM2, glycolysis, inflammasome, PKM2, smilax glabra flavonoids

## Abstract

**Background:**

Nonalcoholic steatohepatitis (NASH) is a prevalent chronic liver disease characterized by hepatic steatosis, persistent inflammation and progressive liver damage. *Smilax glabra* flavonoids (SGF), the major bioactive constituents of *Smilax glabra Roxb*., exhibit potent antioxidant and anti-inflammatory activities. Nevertheless, their therapeutic role and molecular basis in NASH remain elusive.

**Methods:**

This study aimed to investigate the protective effects of SGF against high-fat diet (HFD)-induced NASH in mice, with emphasis on PKM2-mediated glycolysis and AIM2 inflammasome activation. C57BL/6J mice were fed an HFD for 20 weeks to establish a NASH model and concurrently treated with SGF (30 or 90 mg/kg/day) or empagliflozin (EMPA). Comprehensive assessments, including biochemical detection, histological staining and molecular biological verification, were performed. *In vitro*, FFA-challenged AML-12 hepatocytes and LPS-stimulated RAW264.7 macrophages were adopted. siRNA-mediated knockdown, lentiviral overexpression, and cell co-culture models were further applied to validate the underlying mechanisms.

**Results:**

SGF markedly attenuated HFD-induced obesity, dyslipidemia, hepatic steatosis, fibrosis and hepatocellular damage, mitigated hepatic oxidative stress and restored mitochondrial structure integrity. Mechanistically, SGF restrained glycolytic metabolism by downregulating the expression of HK2, p-PKM2, and LDHA and by reducing pyruvate and lactate production. Besides, SGF mitigated AIM2 inflammasome activation and decreased the levels of AIM2, ASC, caspase-1, and IL-1β. Mechanistic validation confirmed that PKM2 was abundantly expressed in macrophages, and PKM2 overexpression partially abrogated the inhibitory effects of SGF on glycolysis and AIM2 inflammasome signaling. Co-culture assays further demonstrated that PKM2 overexpression in RAW264.7 macrophages exacerbated lipid deposition, oxidative stress, glycolysis, and AIM2 inflammasome activation in AML-12 hepatocytes, thereby counteracting the hepatoprotective effects of SGF.

**Conclusion:**

SGF effectively ameliorates HFD-induced NASH by reversing metabolic disturbance, relieving oxidative stress and liver injury, and suppressing PKM2-dependent glycolysis and AIM2 inflammasome activation. Macrophage PKM2 acts as a key mediator of pathogenic macrophage-hepatocyte crosstalk, which is critical for the hepatoprotective effects of SGF against NASH. Overall, our study findings highlight SGF as a promising therapeutic option for this specific population.

## 1 Introduction

Non-alcoholic steatohepatitis (NASH) is a severe and progressive subtype of nonalcoholic fatty liver disease (NAFLD), pathologically characterized by excessive hepatic steatosis, persistent inflammatory infiltration, hepatocyte damage and apoptosis. Advanced NASH further drives the development of liver fibrosis and even irreversible cirrhosis (1). Epidemiological investigations have demonstrated that NASH accounts for 3–5% of all NAFLD cases worldwide (2, 3). Patients diagnosed with NASH present a markedly high susceptibility to cardiovascular complications and adverse liver-related outcomes (4, 5). Critically, approximately 20% of NASH patients eventually progress to cirrhosis and hepatocellular carcinoma (HCC). Notably, recent clinical breakthroughs have achieved preliminary progress in NASH pharmacotherapy. The U.S. Food and Drug Administration (FDA) has approved resmetirom (a selective thyroid hormone receptor-β agonist) and semaglutide (a GLP-1 receptor agonist) for the clinical treatment of NASH, providing new therapeutic options for patients (6). Meanwhile, numerous targeted pharmacological agents are currently under preclinical and clinical evaluation for NAFLD/NASH intervention (7, 8). Nevertheless, lifestyle modification, including weight loss, dietary restriction, and physical exercise, remains the mainstream clinical strategy for NASH management (9, 10). It is noteworthy that end-stage cirrhosis and HCC derived from progressive NASH, rather than simple NASH, constitute the primary indications for liver transplantation in current clinical practice. Despite emerging therapeutic advances, high-efficiency, safe, and mechanism-specific drugs for precisely arresting NASH progression are still insufficient. In particular, traditional Chinese medicine (TCM)-derived bioactive compounds lack systematic mechanistic interpretation and high-quality experimental evidence for NASH treatment, highlighting the urgent demand to explore natural candidates and clarify their precise molecular targets.

Given the enormous public health burden and socioeconomic costs caused by the global NASH epidemic, specific and safe therapeutic drugs for NASH are still lacking in clinical practice. Accumulating evidence has demonstrated that metabolic reprogramming, especially enhanced aerobic glycolysis, serves as a core driving event during NASH pathogenesis. The liver is the central organ governing systemic glucose and lipid metabolic homeostasis. Under physiological conditions, hepatocytes generate energy predominantly through mitochondrial oxidative phosphorylation. However, NASH progression triggers robust metabolic reprogramming, shifting hepatocyte energy metabolism from oxidative phosphorylation to aerobic glycolysis, even under normoxic conditions (11). Clinical evidence has confirmed that key glycolysis-related proteins are highly expressed and that glycolytic metabolites, such as pyruvate, exhibit abnormal accumulation in liver tissues from NASH patients (12). Emerging single-cell RNA sequencing and spatial transcriptomic analyses further confirmed the robust enrichment of glycolysis signaling in NAFLD liver lesions, which is mainly restricted to hepatocytes, macrophages and hepatic fibroblasts (13). Multiple rate-limiting enzymes and transporters involved in glycolysis, including hexokinase 2 (HK2), pyruvate kinase muscle 2 (PKM2), phosphofructokinase, liver type (PFKL) and glucose transporter type 1 (GLUT1), are significantly upregulated in NASH, collectively triggering excessive aerobic glycolysis, also known as the Warburg effect.

Dysregulated glycolysis exacerbates hepatic lipid deposition, inflammatory cascade activation and fibrotic progression by modulating *de novo* lipogenesis, pro-inflammatory cytokine secretion and fibrosis-associated protein expression (14–16). Targeted inhibition of abnormal glycolysis has been widely recognized as a promising therapeutic strategy to alleviate NASH-related liver injury. For instance, the glycolytic inhibitor 2-deoxy-d-glucose (2-DG) effectively ameliorates hepatic lipid accumulation, inflammatory infiltration, and fibrosis in NASH animal models (17, 18). As a pivotal rate-limiting enzyme and multifunctional signaling hub in glycolysis, PKM2 not only controls pyruvate generation and energy metabolism reprogramming but also bridges metabolic dysfunction and inflammatory activation (19). Nevertheless, the precise regulatory mechanisms linking PKM2-mediated glycolysis to NASH progression remain incompletely elucidated (20). Importantly, systematic studies focusing on TCM-derived natural compounds targeting PKM2-dependent glycolysis for NASH intervention are still limited, leaving considerable room for further exploration of novel metabolic regulatory agents.

Inflammasomes are vital innate immune sensors that orchestrate inflammatory responses and participate in the pathological processes of multiple autoimmune and metabolic liver diseases (21). Continuous stimulation by free fatty acids and metabolic stress facilitates the assembly and activation of inflammasome complexes in both hepatic parenchymal cells and resident immune cells. The absent in melanoma 2 (AIM2) inflammasome, a classic HIN-200 family member, consists of an N-terminal pyrin domain (PYD) and a C-terminal HIN domain (22). Upon recognition of cytoplasmic dsDNA/mtDNA induced by metabolic stress and mitochondrial damage, the PYD domain of AIM2 recruits and oligomerizes the adaptor protein ASC, further mediating the cleavage and activation of caspase-1. Activated caspase-1 subsequently promotes the maturation and secretion of pro-inflammatory mediators, including interleukin-1β (IL-1β) and IL-18, thereby aggravating hepatocyte injury, macrophage overactivation, and hepatic fibrotic remodeling (23, 24). Accumulating preclinical studies have verified that specific blockade of AIM2 inflammasome activation significantly relieves hepatic inflammatory infiltration and retards NASH fibrotic progression (25–27), indicating that the AIM2 inflammasome serves as a critical and druggable therapeutic target for NASH.

Natural products and TCM have attracted increasing attention for the prevention and treatment of chronic metabolic liver diseases due to their multi-target characteristics, low toxicity and long-term prognostic benefits compared with synthetic chemical drugs. *Smilax glabra* Roxb. is a classic herbal medicine widely used in TCM, and *Smilax glabra* flavonoids (SGF) represent the major bioactive constituents extracted from its dried rhizome. Accumulating pharmacological studies have demonstrated that SGF has multiple biological functions, including anti-inflammation, anti-oxidation, immune regulation, cardiovascular protection, and anti-tumor activity (28–34). Notably, recent research has reported that SGF intervention reduces hepatic lipid accumulation and alleviates oxidative damage in a high-cholesterol diet-induced fatty liver zebrafish model (30), suggesting a potential hepatoprotective role of SGF in metabolic fatty liver disorders. Nevertheless, the preventive and therapeutic efficacy as well as the detailed molecular mechanism of SGF against HFD-induced NASH remain poorly defined.

In the present study, focusing on the two pivotal hepatic cell types (hepatocytes and macrophages), we systematically explored the protective effects of SGF against NASH progression. Specifically, we clarified that SGF ameliorates hepatic glycolytic reprogramming and inflammatory injury via suppressing macrophage PKM2-dependent glycolysis and subsequent AIM2 inflammasome activation through macrophage–hepatocyte crosstalk. This work provides novel experimental evidence and mechanistic insights for SGF as a promising natural therapeutic candidate for NASH intervention.

## 2 Materials and methods

### 2.1 Smilax glabra flavonoid preparation

SGF was prepared from the rhizomes of *Smilax glabra* (Zhejiang Chinese Medical University Medicine Ltd., Hangzhou, China), as described previously (32, 34). Briefly, the rhizomes of *Smilax glabra* were extracted with 60% ethanol at a solid-to-liquid ratio of 1:20 (w/v), followed by purification using HP700 macroporous resin with 60% ethanol as the eluent. The flavonoid-rich fractions were concentrated at 45 °C and lyophilized to obtain SGF. The total flavonoid content of SGF was 98.65% by UV spectrophotometry (using rutin as the standard), and the astilbin content was 25.13% as measured by high-performance liquid chromatography (HPLC, Waters e2695).

### 2.2 Animal experimental protocol

Forty Specific Pathogen-Free (SPF)-grade male C57BL/6J mice (age: 6–8 weeks; weight: 16–24 g) were purchased from Sino-British SIPPR/BK Laboratory Animal Co., Ltd. (certificate number: SCXK, 2023-0009) and maintained in the Laboratory Animal Research Center of Zhejiang Chinese Medical University (SYXK2021-0012). All animal procedures were approved by the Institutional Animal Care and Use Committee (IACUC) of Zhejiang Chinese Medical University (IACUC-20240909-16) and conducted in accordance with relevant guidelines. After one week of acclimatization, the mice were randomly divided into five groups based on body weight and treated continuously for 20 weeks. Mice in the control group were fed a low-fat diet (LFD, providing 10% kcal from fat), while mice in the other four groups were fed a high-fat diet (HFD, providing 60% kcal from fat; Research Diets PD6001) to induce NASH. Concurrently, mice in the treatment groups were administered SGF at doses of 30 mg/kg/day or 90 mg/kg/day (SGF-L or SGF-H, respectively) (32) or EMPA at 10 mg/kg/day (35) via oral gavage once daily. The dosage of SGF was determined based on the mouse-rat dosage conversion coefficient and previous studies. On the final day of treatment, mice were fasted for 12 h, blood samples were collected from the orbital venous plexus, and mice were euthanized by cervical dislocation.

### 2.3 Biochemical indicators detection

After 20 weeks of treatment, mice were fasted for 12 h prior to blood collection from the orbital venous plexus. Serum levels of total cholesterol (TC), triglycerides (TG), high-density lipoprotein cholesterol (HDL-C), low-density lipoprotein cholesterol (LDL-C), glucose (GLU), lactate dehydrogenase (LDH), aspartate aminotransferase (AST), and alanine aminotransferase (ALT) were measured using an automated biochemistry analyzer (Hitachi 3100). Liver tissue levels of TC, TG, LDH, superoxide dismutase (SOD), glutathione (GSH), malondialdehyde (MDA), and catalase (CAT) were measured using commercially available kits (Nanjing Jiancheng Bioengineering Institute, China). Serum levels of tumor necrosis factor-α (TNF-α) and interleukin-1β (IL-1β) were determined using ELISA kits (ABclonal Biotech Co., Ltd, China).

### 2.4 Histopathological analysis

Liver tissue was fixed in 4% paraformaldehyde for 24 h, embedded in paraffin, and sectioned at a thickness of 4μm. Hematoxylin-eosin (H&E) staining was performed for general histopathology evaluation, and Masson’s trichrome staining was used to assess collagen deposition. For lipid distribution and reactive oxygen species (ROS) detection, liver tissues were rapidly frozen, embedded in OCT compound, and cryosectioned into 8-μm-thick slices. These sections were stained with Oil Red O to visualize lipid droplets and with dihydroethidium (DHE) to detect intracellular ROS. All sections were digitally scanned using a NanoZoomer 2.0-RS whole slide scanner (Hamamatsu Photonics, Hamamatsu, Japan) or a VS120-S6-W fluorescence digital pathology slide scanner system (Olympus Corporation, Tokyo, Japan).

### 2.5 Immunofluorescence staining

Paraffin sections were dewaxed in xylene and rehydrated through a graded ethanol series. Antigen retrieval was performed using EDTA buffer, followed by the inactivation of endogenous peroxidase with 3% H_2_O_2_. The sections were blocked with 3% BSA for 30 minutes and then incubated with the primary antibody at 4 °C overnight. After washing, sections were incubated with the secondary antibody at 37 °C, followed by incubation with CY3 or FITC under light-protected conditions (Boster Biological Technology, China). Finally, the sections were mounted with an antifade mounting medium containing DAPI (Abcam, China) to stain cell nuclei. Images were acquired using a VS120-S6-W fluorescence digital pathology slide scanner system (Olympus Corporation, Tokyo, Japan).

### 2.6 Cell culture and treatment

The mouse liver cell line AML-12 cells (Procell Life Science & Technology, Wuhan, China) and murine macrophage cell line RAW 264.7 (Chinese Academy of Sciences Cell Bank, Beijing, China) were cultured in DMEM medium containing 10% fetal bovine serum and 1% penicillin-streptomycin and maintained in an incubator at 37 °C with 5% CO_2_. To establish an *in vitro* model of hepatic steatosis, the AML-12 cells were treated with 1 mM free fatty acids (FFAs) (OA: PA molar ratio = 2:1) for 24 h as previously described (26). RAW264.7 cells were stimulated with lipopolysaccharide (LPS) to induce M1 macrophage polarization (36).

### 2.7 Plasmid and small interfering RNA (siRNA) transfection

Targeting siRNAs and overexpression plasmids were designed and synthesized by GenePharma (Shanghai, China). Cells were seeded in six-well plates, and when the cell density reached 40%, Lipofectamine 3000 (Invitrogen, USA) was used to transfect cells with siRNAs and plasmids in serum-free medium according to the manufacturer’s instructions. The siRNA sequences are summarized in **Supplementary Table 1**.

### 2.8 Lentivirus transduction

For lentiviral transfection, the RAW264.7 cells were divided into the PKM2 overexpression (PKM2^−OE^) group and the empty-vector lentiviral (LV^−Con^) group (GenePharma, China). Cells were infected at a density of 40% with a multiplicity of infection (MOI) of 80 and incubated for 24 hours. After discarding the lentivirus-containing medium, cells were further cultured for 48 hours. At 72 hours post-transfection, puromycin (10μg/mL) (Gene, China) was added to select successfully transfected cells. PKM2 expression was detected by Western blotting.

### 2.9 Oil red O staining

The AML-12 cells were fixed with 4% paraformaldehyde for 15 minutes, stained with Oil Red O for 30 minutes and then differentiated with 60% isopropanol prior to imaging under an inverted microscope.

### 2.10 Lactate and pyruvate assays

Lactate and pyruvate levels in liver tissues or cell culture supernatants were measured using commercial assay kits (Nanjing Jiancheng, China). Tissue samples were homogenized in physiological saline, while cell culture supernatants were used directly without pretreatment. Following the manufacturer’s instructions, tissue homogenates (or cell culture supernatants) were deproteinized and then incubated with the corresponding assay reagents. Absorbance was measured at 530 nm (for lactate) or 505 nm (for pyruvate) using a microplate reader.

### 2.11 Measurement of mitochondrial superoxide

Mitochondrial superoxide levels were measured using MitoSOX (MedChemExpress, USA) to evaluate the level of mitochondrial oxidative stress. Images were captured under an inverted fluorescence microscope.

### 2.12 Quantitative real-time PCR analysis

Total RNA was extracted from liver tissue or cells using TRIzol (Takara, Japan) and quantified, and cDNA was synthesized using the PrimeScript RT Master Mix (Takara, Japan). qRT-PCR was performed using SYBR Green (Vazyme, China) and gene-specific primers. All primer sequences (**Supplementary Table 2**) were designed and synthesized by the Bioengineering Company (Shanghai, China). Gene expression levels were calculated using the ΔΔCt method, with β-actin as the internal reference gene for normalization.

### 2.13 Western blot analysis

The total protein was extracted from liver tissue or cells using a total protein extraction kit (Keygen Biotech, China), and the concentration of the protein was measured using a BCA assay kit (Biosharp, China). Electrophoresis was then performed, followed by transfer onto a nitrocellulose (NC) membrane (Merck, Germany). The membranes were blocked for 2 hours and then incubated with specific primary antibodies (**Supplementary Table 3**) overnight at 4 °C. After incubation with species-matched IRDye-conjugated secondary antibodies (LI-COR Biosciences, NE, USA), the membrane was visualized using an Odyssey infrared imaging system (LI-COR Biosciences, NE, USA), and the results were analyzed using ImageJ.

### 2.14 Statistical analysis

Data were presented as the mean ± standard deviation (SD), with ‘n’ representing the number of independent biological replicates (i.e., animals or cell culture experiments). Normality and homogeneity of variances were assessed using the Shapiro-Wilk and Levene test, respectively. For multiple group comparisons, one-way ANOVA followed by Dunnett’s *post hoc* test was used for parametric data, and the Kruskal-Wallis test followed by Dunn’s *post hoc* test was applied for non-parametric data. A *p*-value < 0.05 was considered statistically significant.

### 2.15 Supplementary methods

The detailed procedures for the supplementary hydroxyproline assays, fluorescence staining of dsDNA and mitotracker, and Co-immunoprecipitation assay are described in the **Supplementary Materials** (**Supplementary Methods S1**–**S3**).

## 3 Results

### 3.1 SGF ameliorates obesity and dyslipidemia in HFD-induced murine NASH model

**Figure 1:**
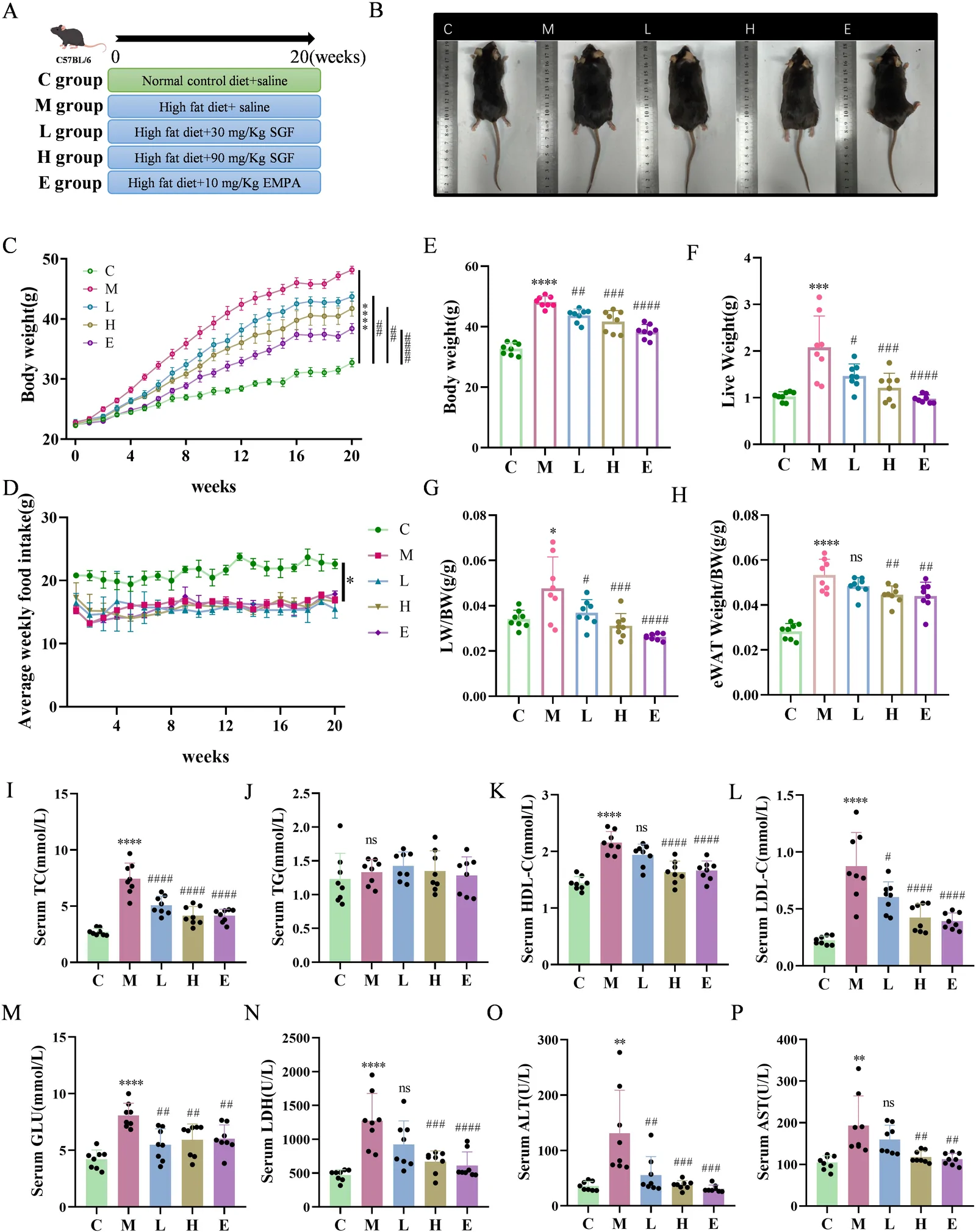
SGF ameliorates obesity and dyslipidemia in HFD-fed mice. **(A)** Schedule of the experimental period. **(B)** Macroscopic images. **(C)** Body weight changes in mice during the administration period. **(D)** Food intake. **(E)** The body weight of the mice at the end of the experiment. **(F)** Liver weight of mice. **(G)** Liver index. **(H)** Epididymal white adipose tissue index. **(I–P)** Serum TC, TG, HDL-C, LDL-C, GLU, LDH, ALT and AST levels were measured using an automatic biochemical analyzer. Data are presented as the mean ± SD (n = 8). *p < 0.05, **p < 0.01, ***p < 0.001, ****p < 0.0001 vs. control; ^#^*p* < 0.05, ^##^*p* < 0.01, ^###^*p* < 0.001, ^####^*p* < 0.0001 vs. model.

To investigate the preventive effects of SGF on NASH, we established a 20-week HFD-induced murine NASH model, which included low- and high-dose SGF treatment groups (30 and 90 mg/kg/day, SGF-L/SGF-H) and a positive control group treated with EMPA (10mg/kg/day) (**Figure 1A**). Representative body morphology and hair condition of the mice in each group are presented in **Figure 1B**. Our findings revealed that SGF-L/SGF-H and EMPA treatment significantly attenuated HFD-induced body weight gain (**Figure 1C**). Food intake in grams was significantly higher in the normal control group compared with the model group; however, there were no significant differences among the various HFD-fed groups. (**Figure 1D**). Compared with the HFD group (Model, M), SGF-L/SGF-H and EMPA markedly decreased the final body weight (**Figure 1E**), liver weight (**Figure 1F**), and liver index (**Figure 1G**). Notably, SGF-H and EMPA also significantly inhibited visceral fat accumulation (**Figure 1H**).

Serum biochemical analysis revealed that SGF ameliorated HFD-induced dyslipidemia: SGF-L/SGF-H and EMPA attenuated the HFD-induced elevations in TC, HDL-C, and LDL-C (**Figures 1I–L**). Serum glucose (GLU) assays demonstrated that SGF-L/SGF-H and EMPA alleviated HFD-induced glucose metabolism disorders (**Figure 1M**). Moreover, SGF-H and EMPA suppressed the HFD-induced increase in LDH levels (**Figure 1N**). Furthermore, serum biochemical results confirmed SGF’s hepatoprotective effects: compared with group M, ALT and AST levels were significantly lower in the SGF-L/SGF-H and EMPA groups, indicating improved liver function (**Figures 1O, P**). Collectively, these results suggest that SGF reduces HFD-induced fat accumulation, ameliorates lipid/glucose metabolism disorders, and mitigates liver injury in mice.

### 3.2 SGF alleviates HFD-induced hepatic steatosis and fibrosis in mice

**Figure 2:**
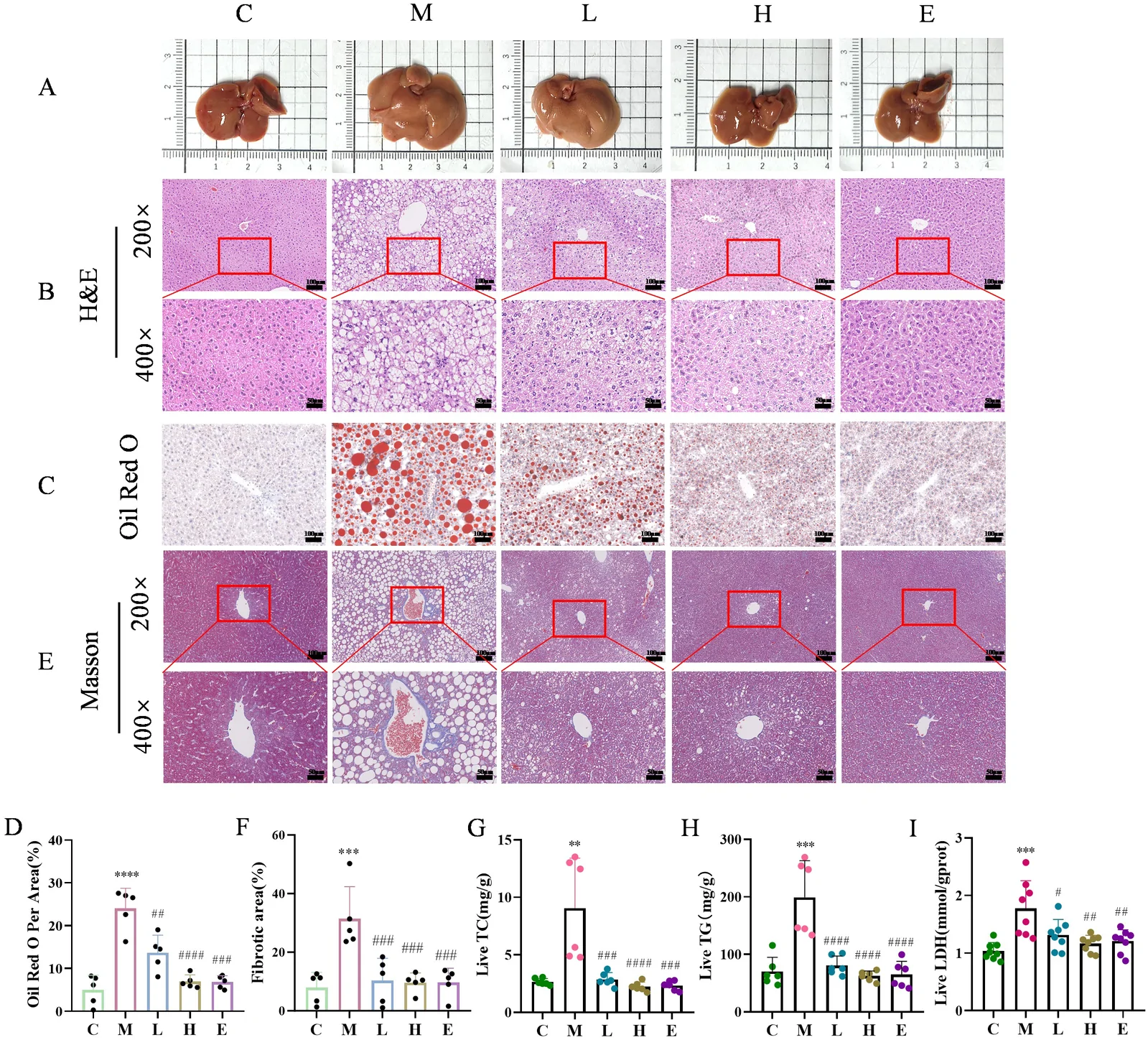
SGF alleviates hepatic steatosis and liver injury in HFD-fed mice. **(A)** Liver macroscopic images. **(B)** H&E staining. **(C)** Oil Red O staining. **(D)** Oil Red O staining area quantification. **(E, F)** Masson's staining and quantification of fibrotic area. **(G–I)** Live TC, TG, and LDH. Data are presented as the mean ± SD (n = 5-6). *p < 0.05, **p < 0.01, ***p < 0.001, ****p < 0.0001 vs. control; ^#^*p* < 0.05, ^##^*p* < 0.01, ^###^*p* < 0.001, ^###^#*p* < 0.0001 vs. model.

Macroscopic examination of mouse liver tissues revealed striking intergroup pathological differences: the HFD model group displayed typical fatty liver features, characterized by hepatomegaly, pale discoloration, and a greasy texture, while these abnormalities were markedly ameliorated in the SGF-L/SGF-H and EMPA treatment groups (**Figure 2A**). The H&E staining results indicated that, compared to the control group, hepatocytes in the model group displayed obvious structure disorganization, severe steatosis, and abundant cytoplasmic lipid vacuoles; SGF-L/SGF-H or EMPA treatment significantly attenuated these pathological abnormalities, as evidenced by reduced steatosis areas and restored hepatocellular arrangement (**Figure 2B**). Supplementary Oil Red O staining further validated that SGF-L/SGF-H and EMPA markedly reduced lipid droplet accumulation in liver tissues (**Figures 2C, D**). Masson’s staining demonstrated prominent collagen deposition around the central vein in the model group’s liver tissues, whereas the fibrotic area was significantly diminished in the SGF-L/SGF-H and EMPA groups (**Figures 2E, F**). Hydroxyproline assay also confirmed that SGF and EMPA alleviated hepatic fibrosis (**Supplementary Figure 2A**). Quantification of hepatic TG, TC, and LDH levels confirmed that SGF-L/SGF-H and EMPA effectively corrected HFD-induced hepatic lipid metabolic disorders and alleviated hepatocellular injury (**Figures 2G–I**). Collectively, these results indicated that SGF exerts a potent preventive effect against HFD-induced fatty liver injury in mice.

### 3.3 SGF mitigates HFD-induced hepatic oxidative stress in mice

**Figure 3:**
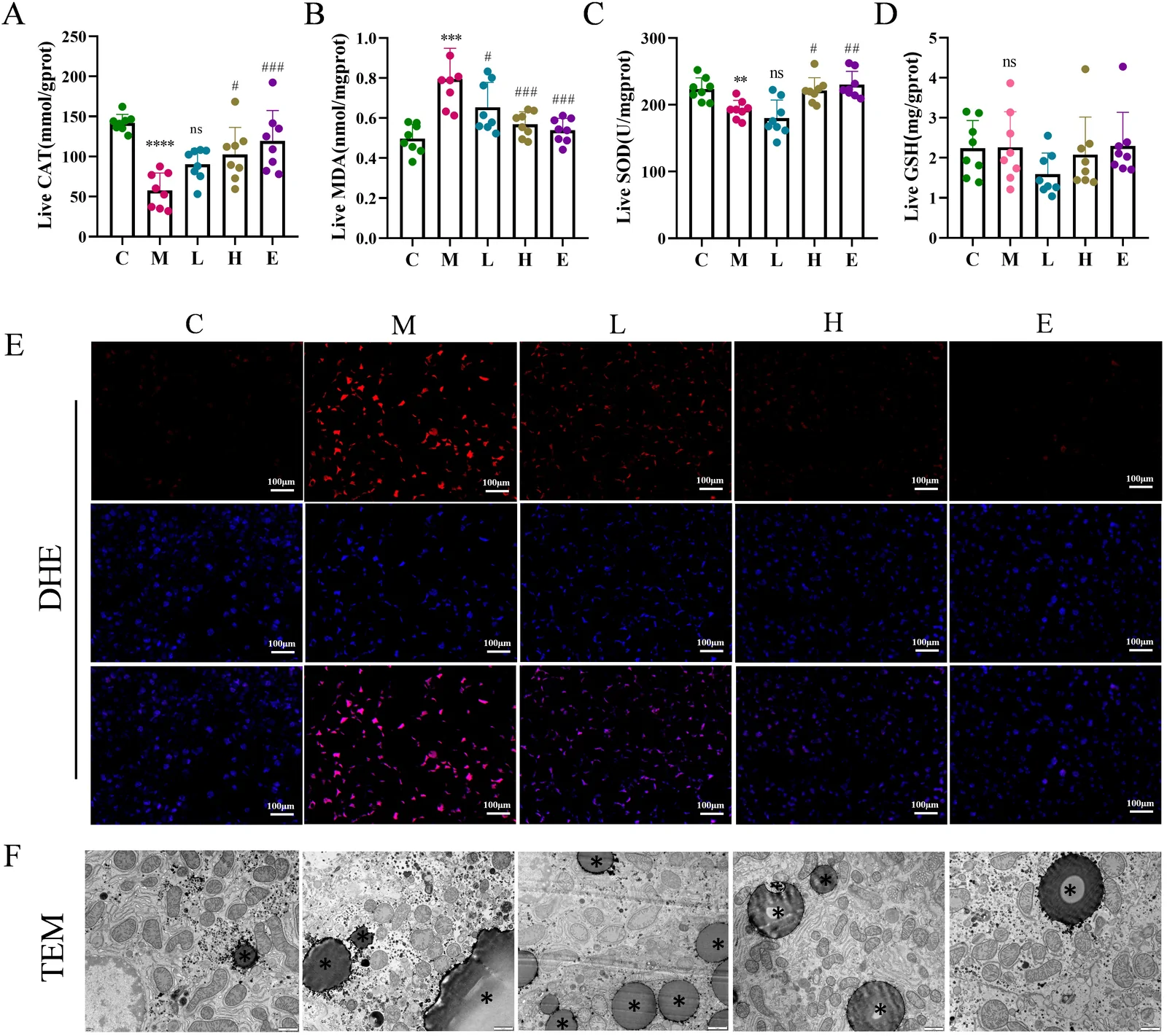
SGF mitigates hepatic oxidative stress in HFD-fed mice. **(A–D)** Hepatic levels of CAT, MDA, SOD, and GSH. **(E)** Representative images of DHE staining. **(F)** Representative TEM images, * represent lipid droplets. Data are presented as the mean ± SD (n = 8). *p < 0.05, **p < 0.01, ***p < 0.001, ****p < 0.0001 vs. control; ^#^*p* < 0.05, ^##^*p* < 0.01, ^###^*p* < 0.001, ^####^*p* < 0.0001 vs. model.

We next evaluated key biomarkers associated with oxidative stress in each group. In the HFD model group, the enzymatic activities of SOD and CAT were significantly decreased; in contrast, SGF-H and EMPA treatment markedly upregulated these antioxidant enzyme activities (**Figures 3A, C**). MDA levels, a marker of lipid peroxidation, were significantly increased in the model group, while SGF-L/SGF-H and EMPA both abrogated these elevation (**Figure 3B**). No significant differences in GSH levels were observed among all groups (**Figure 3D**). DHE staining revealed that intracellular reactive oxidative species (ROS) accumulation was significantly reduced in the SGF-L, SGF-H, and EMPA groups relative to the model group (**Figure 3E**). Transmission electron microscopy (TEM) analysis showed prominent mitochondrial swelling, cristae fragmentation, and large lipid droplet accumulation in hepatocytes of the model group; in contrast, the SGF-H and EMPA groups exhibited marked improved mitochondrial morphology, accompanied by a significant decrease in lipid droplet deposition (**Figure 3F**). These results confirm that SGF possessed robust antioxidant activity and effectively mitigates HFD-induced hepatic oxidative stress in mice.

### 3.4 SGF inhibits HFD-induced upregulation of hepatic glycolysis and AIM2 inflammasome activation in mice

**Figure 4:**
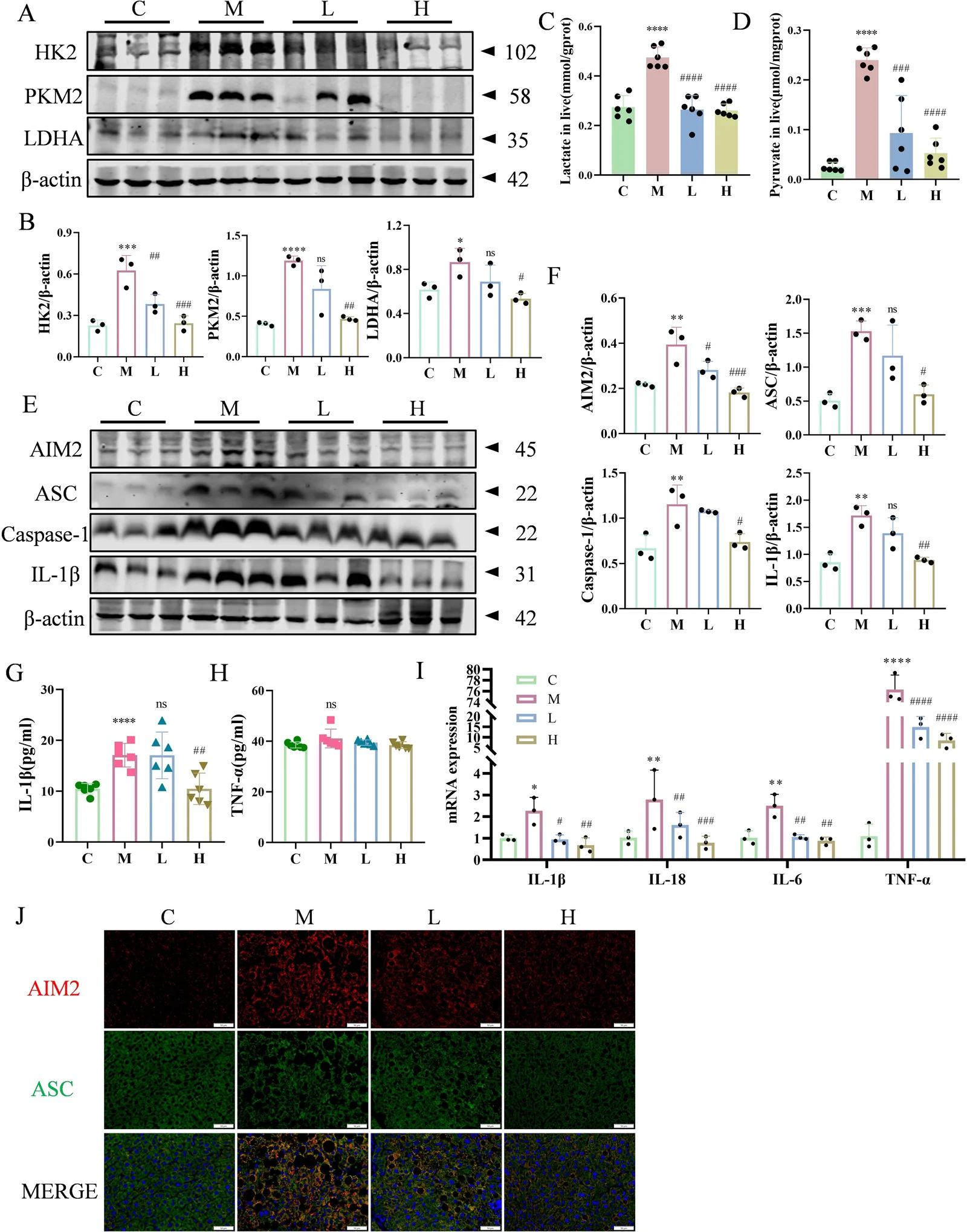
SGF inhibits hepatic glycolysis and AIM2 inflammasome activation in HFD-fed mice. **(A, B)** Protein expression of HK2, PKM2 and LDHA in the liver. **(C, D)** Lactate and pyruvate expression in the liver. **(E, F)** Protein expression of AIM2, ASC, Caspase-1 and IL-1β in the liver. **(G, H)** ELISA measurement of serum IL-1β and TNF-α levels. **(I)** mRNA expression levels of IL-1β, IL-18, IL-6, and TNF-α. **(J)** AIM2 inflammasome fluorescence colocalization images. Data are presented as the mean ± SD (n = 3-6). *p < 0.05, **p < 0.01, ***p < 0.001, ****p < 0.0001 vs. control; ^#^*p* < 0.05, ^##^*p* < 0.01, ^###^*p* < 0.001, ^####^*p* < 0.0001 vs. model.

It is well-established that glycolysis is upregulated in hepatic NASH tissues (14). We next examined the expression of glycolysis-related proteins and found that, compared with the control group, the HFD model group showed significantly increased protein levels of HK2, PKM2, and LDHA, and these upregulations were significantly inhibited by SGF-H treatment (**Figures 4A, B**). In addition, the key glycolytic metabolites (pyruvate and lactate) were significantly elevated in the model group, and SGF treatment markedly reduced their hepatic levels (**Figures 4C, D**). SGF also exerted a prominent anti-inflammatory effect in the liver. Western blot analysis showed that the protein expression of AIM2 inflammasome-related components (AIM2, ASC, Caspase-1, and IL-1β) was significantly upregulated in the HFD model group, and SGF-H treatment markedly downregulated their expression (**Figures 4E, F**). ELISA results further showed that SGF-H significantly inhibited the HFD-induced elevation of serum IL-1β levels, with no significant alteration in TNF-α levels across groups (**Figures 4G, H**). qRT-PCR analysis revealed that the hepatic mRNA expression of pro-inflammatory cytokines (IL-1β, IL-18, IL-6, and TNF-α) was significantly upregulated in the HFD model group, and SGF treatment inhibited their transcription (**Figure 4I**). Fluorescence colocalization assays demonstrated that AIM2 inflammasome expression was significantly enhanced in the HFD model group, and SGF treatment inhibited its hepatic expression, with the inhibitory effect gradually increasing as the concentration rose (**Figure 4J**). Overall, these results suggest that SGF inhibits HFD-induced upregulation of hepatic glycolysis and AIM2 inflammasome in murine liver.

### 3.5 SGF suppresses FFA-induced glycolysis activation and AIM2 inflammasome upregulation in AML-12 cells

**Figure 5:**
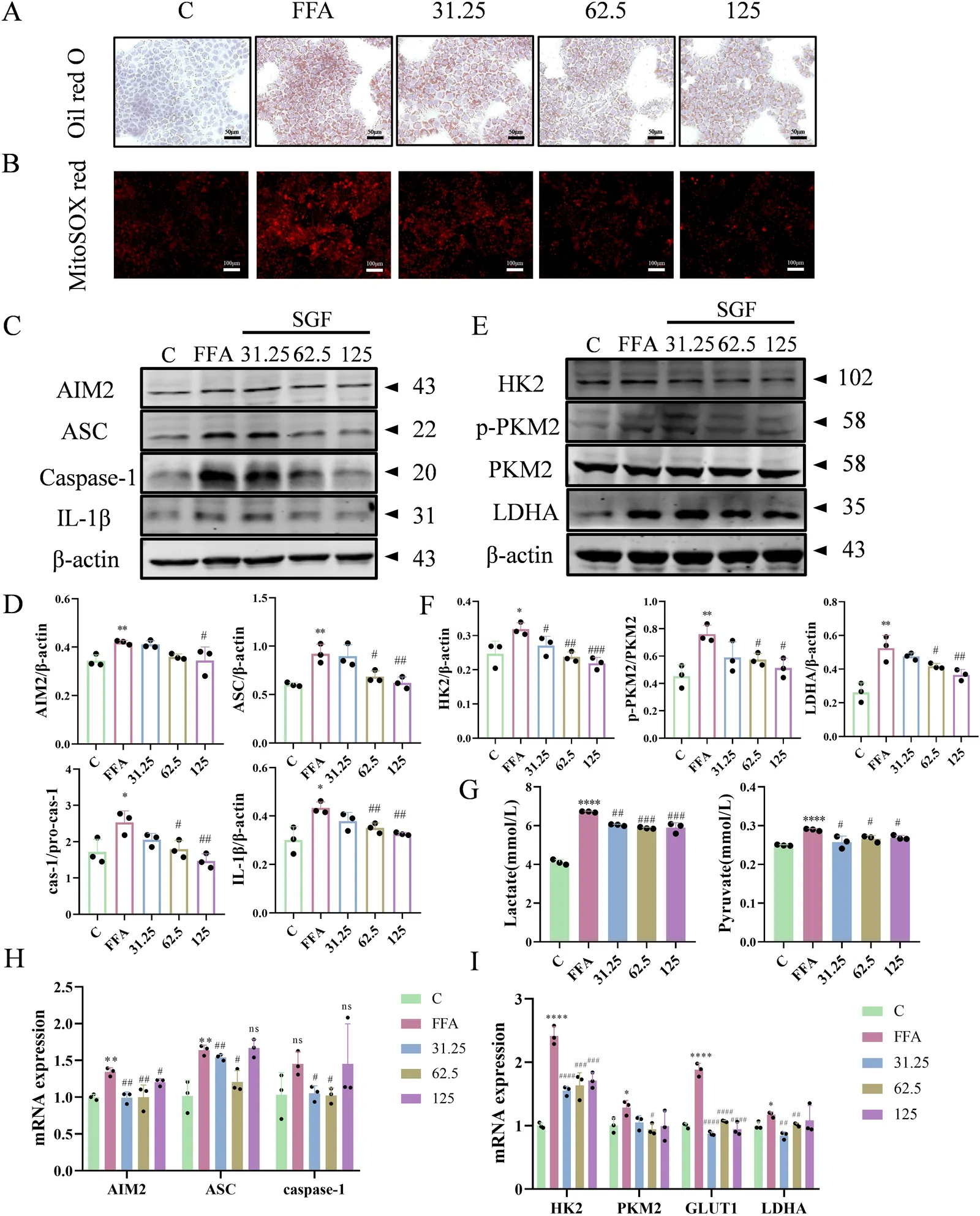
SGF suppresses FFA-induced glycolysis activation and AIM2 inflammasome upregulation in AML-12 cells. **(A)** Oil Red O staining. **(B)** MitoSOX Red staining. **(C, D)** Protein expression of AIM2, ASC, Caspase-1 and IL-1β. **(E, F)** Protein expression of HK2, p-PKM2, PKM2 and LDHA. **(G)** Levels of lactate and pyruvate expression in the cell supernatant. **(H)** mRNA expression levels of AIM2, ASC and caspase-1. **(I)** mRNA expression levels of HK2, PKM2, GLUT1 and LDHA. Data are presented as the mean ± SD (n = 3). *p < 0.05, **p < 0.01, ***p < 0.001, ****p < 0.0001 vs. C; ^#^*p* < 0.05, ^#^#*p* < 0.01, ^###^*p* < 0.001, ^####^*p* < 0.0001 vs. FFA.

We further validated the *in vivo* findings in mouse hepatocyte AML-12 cells *in vitro*. We first identified non-cytotoxic concentrations of SGF via cytotoxicity assays, and selected 31.25, 62.5, and 125 μg/mL for subsequent experiments (**Supplementary Figure 1A**). Oil Red O staining showed that FFA stimulation significantly increased intracellular lipid droplet accumulation in AML1–12 cells, while 62.5 and 125 μg/mL treatment markedly reduced this accumulation (**Figure 5A**). Similarly, MitoSOX Red staining revealed that SGF treatment exerted a significant antioxidant effect in FFA-stimulated AML-12 cells (**Figure 5B**). Consistently, dsDNA staining results further confirmed that SGF effectively suppressed FFA-induced cytosolic dsDNA leakage (**Supplementary Figure 2B**). Western blot analysis confirmed the anti-inflammatory activity of SGF, showing that SGF inhibited FFA-induced upregulation of AIM2 inflammasome-related proteins (AIM2, ASC, caspase-1, and IL-1β) (**Figures 5C, D**). Consistent with the *in vivo* findings, the inhibitory effect of SGF on glycolysis was confirmed in this *in vitro* model. Specifically, Western blot analysis revealed that SGF treatment significantly downregulated the expression of HK2, p-PKM2, and LDHA (**Figures 5E, F**), which was accompanied by a marked reduction in extracellular lactate and pyruvate levels (**Figure 5G**). The mRNA expression profiles of AIM2 inflammasome-related genes and glycolysis-related genes were consistent with the protein expression results (**Figures 5H, I**). These *in vitro* results confirm that SGF pretreatment reduces FFA-induced intracellular lipid droplet accumulation, exerts robust antioxidant and anti-inflammatory effects, and inhibits aberrant activation of glycolysis in hepatocytes.

### 3.6 PKM2 knockdown inhibits FFA-induced AIM2 inflammasome activation in AML-12 cells

**Figure 6:**
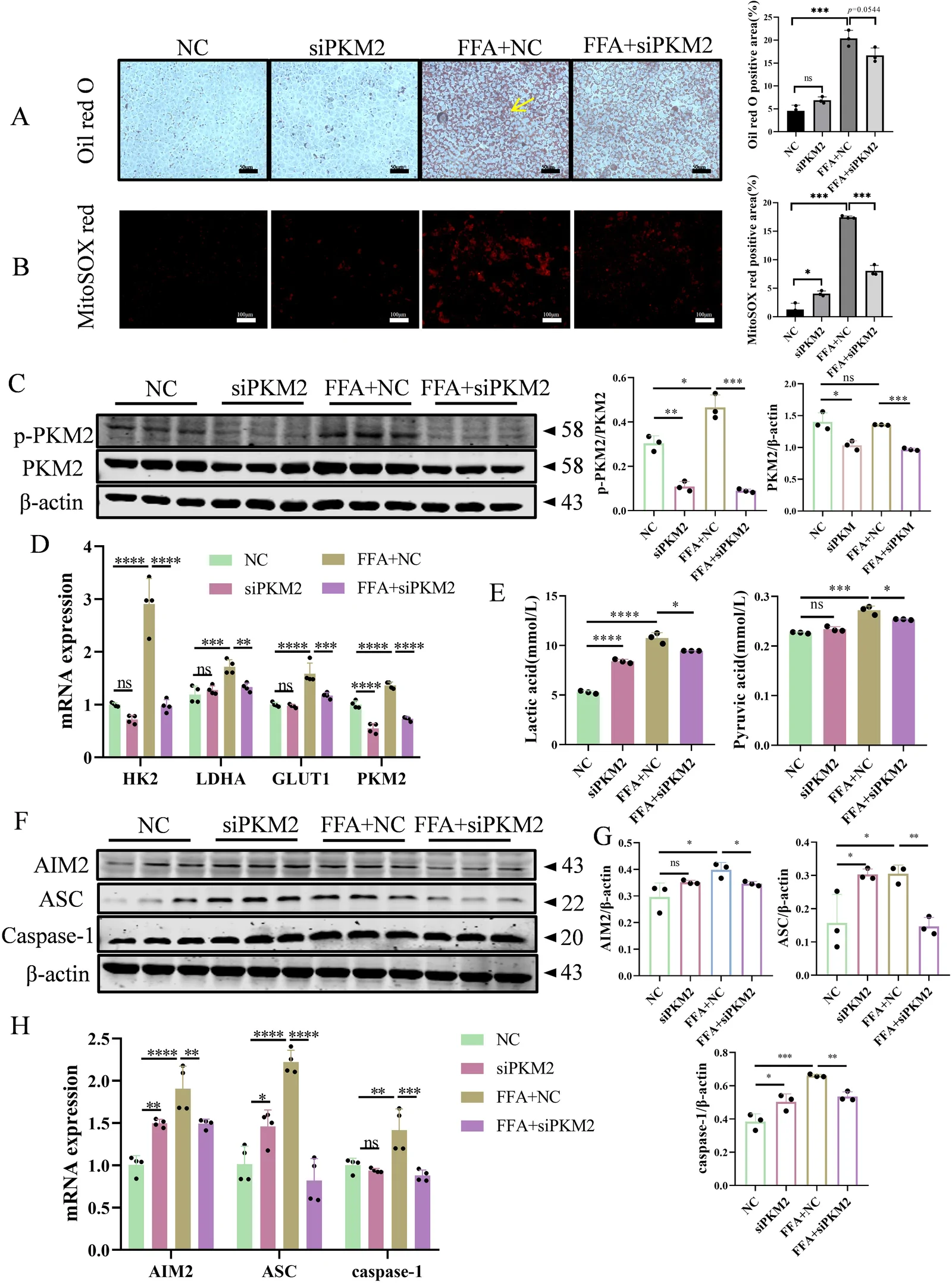
PKM2 knockdown inhibits FFA-induced AIM2 inflammasome activation in AML-12 cells. **(A)** Oil Red O staining, yellow arrows indicate large lipid droplets. **(B)** MitoSOX Red staining. **(C)** Protein expression of p-PKM2 and PKM2. **(D)** mRNA expression levels of HK2, PKM2, GLUT1, and LDHA. **(E)** Levels of lactate and pyruvate expression in the cell supernatant. **(F, G)** Protein expression of AIM2, ASC, and Caspase-1. **(H)** mRNA expression levels of AIM2, ASC, and caspase-1. Data are presented as the mean ± SD (n = 3-4). *p < 0.05, **p < 0.01, ***p < 0.001, ****p < 0.0001.

Pyruvate Kinase M2 (PKM2), a key rate-limiting metabolic enzyme in the glycolytic pathway, is closely associated with obesity and NAFLD (37). To explore the regulatory role of PKM2 in NASH pathogenesis, we transfected AML-12 cells with PKM2-targeting siRNA to knock down PKM2 expression. Oil Red O staining revealed a mild reduction in FFA-induced intracellular lipid accumulation following PKM2 silencing, although this change did not reach statistical significance (P = 0.0544). Morphologically, large lipid droplets appeared to break down into smaller lipid droplets upon PKM2 knockdown (**Figure 6A**). MitoSOX Red staining revealed that PKM2 knockdown significantly attenuated the FFA-induced elevation of mitochondrial ROS levels (**Figure 6B**). Meanwhile, Western blot analysis showed that PKM2 knockdown significantly inhibited PKM2 phosphorylation (**Figure 6C**) and downregulated the mRNA expression of glycolysis-related genes (**Figure 6D**). The levels of glycolytic end products (lactate and pyruvate) in the cell supernatant also exhibited a downward trend (**Figure 6E**). PKM2 knockdown significantly suppressed the activation of the AIM2 inflammasome in FFA-stimulated AML-12 cells. Specifically, compared to the FFA-treated group, genetic silencing of PKM2 markedly reduced both the protein and mRNA expression levels of AIM2, ASC, and caspase-1 (**Figures 6F–H**). These results collectively indicated that PKM2 exerts a regulatory role in FFA-induced hepatocellular stress responses and is a critical regulator of AIM2 inflammasome activation in hepatocytes.

### 3.7 SGF exerts pharmacological effects in AML-12 cells independent of PKM2 overexpression

**Figure 7:**
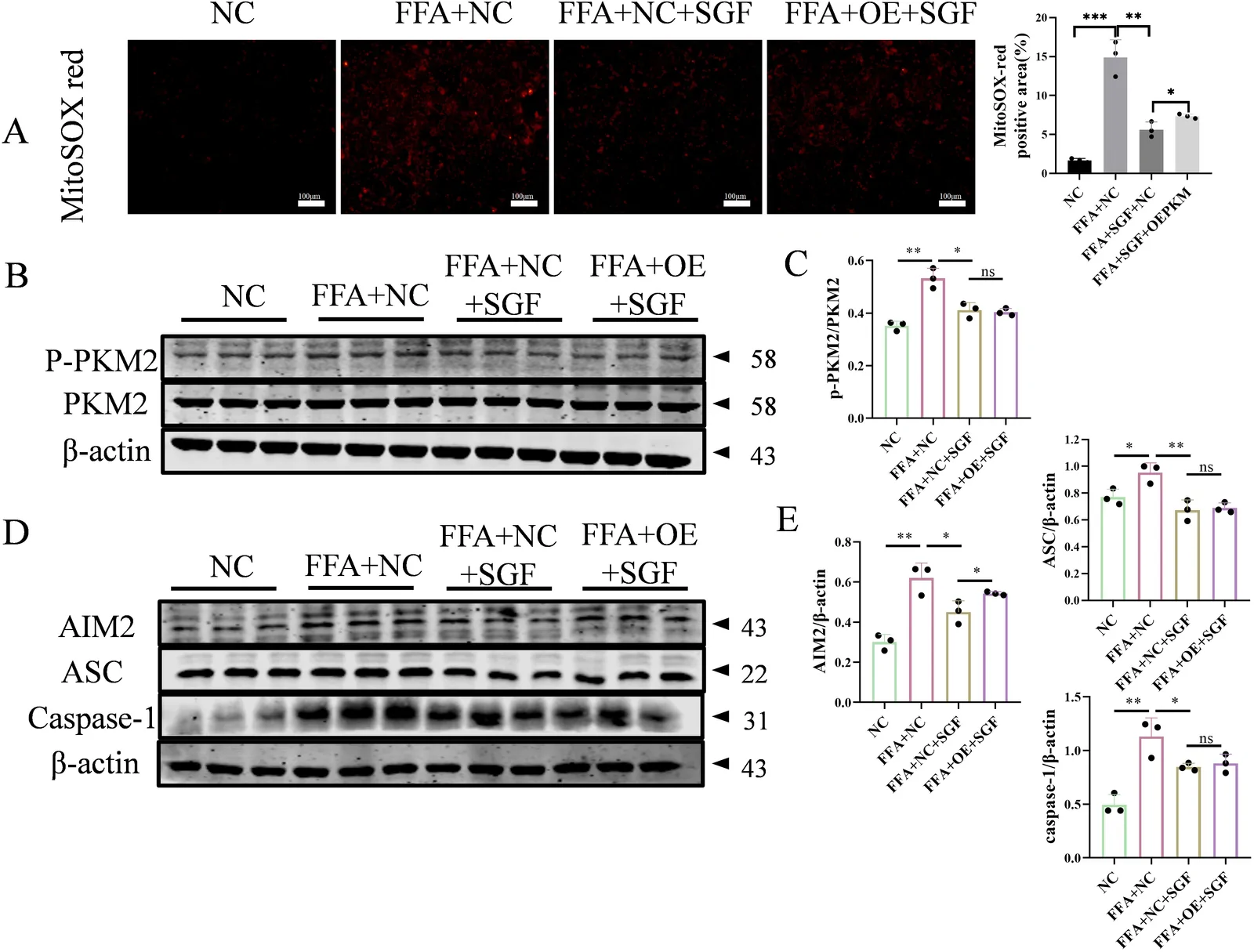
PKM2 overexpression fails to abrogate the pharmacological effects of SGF in AML-12 cells. **(A)** MitoSOX Red staining. **(B, C)** Protein expression of p-PKM2 and PKM2. **(D, E)** Protein expression of AIM2, ASC and Caspase-1. Data are presented as the mean ± SD (n = 3). *p < 0.05, **p < 0.01, ***p < 0.001, ****p < 0.0001.

Having confirmed that PKM2 knockdown inhibits AIM2 inflammasome activation in AML-12 cells, we next investigated whether PKM2 overexpression could abrogate the pharmacological effects of SGF in these cells. We first validated plasmid transfection efficiency via fluorescence microscopy, and confirmed elevated PKM2 protein levels using Western blotting (**Supplementary Figure 2C**). MitoSOX Red staining indicated that PKM2 overexpression caused a slight increase in mitochondrial superoxide levels in FFA-stimulated AML-12 cells (**Figure 7A**). However, Western blot analysis showed that this moderate PKM2 overexpression did not notably reverse the inhibitory effect of SGF on PKM2 phosphorylation (**Figures 7B, C**), and exerted no apparent effect on AIM2 inflammasome-related protein expression (**Figures 7D, E**). Given that PKM2 is highly expressed in macrophages (37), we established an LPS-induced inflammation model in murine macrophage RAW264.7 cells to explore the role of PKM2 in NASH and the possible mechanism underlying SGF’s effects.

### 3.8 SGF inhibits LPS-induced glycolysis and AIM2 inflammasome activation in RAW264.7 macrophages

**Figure 8:**
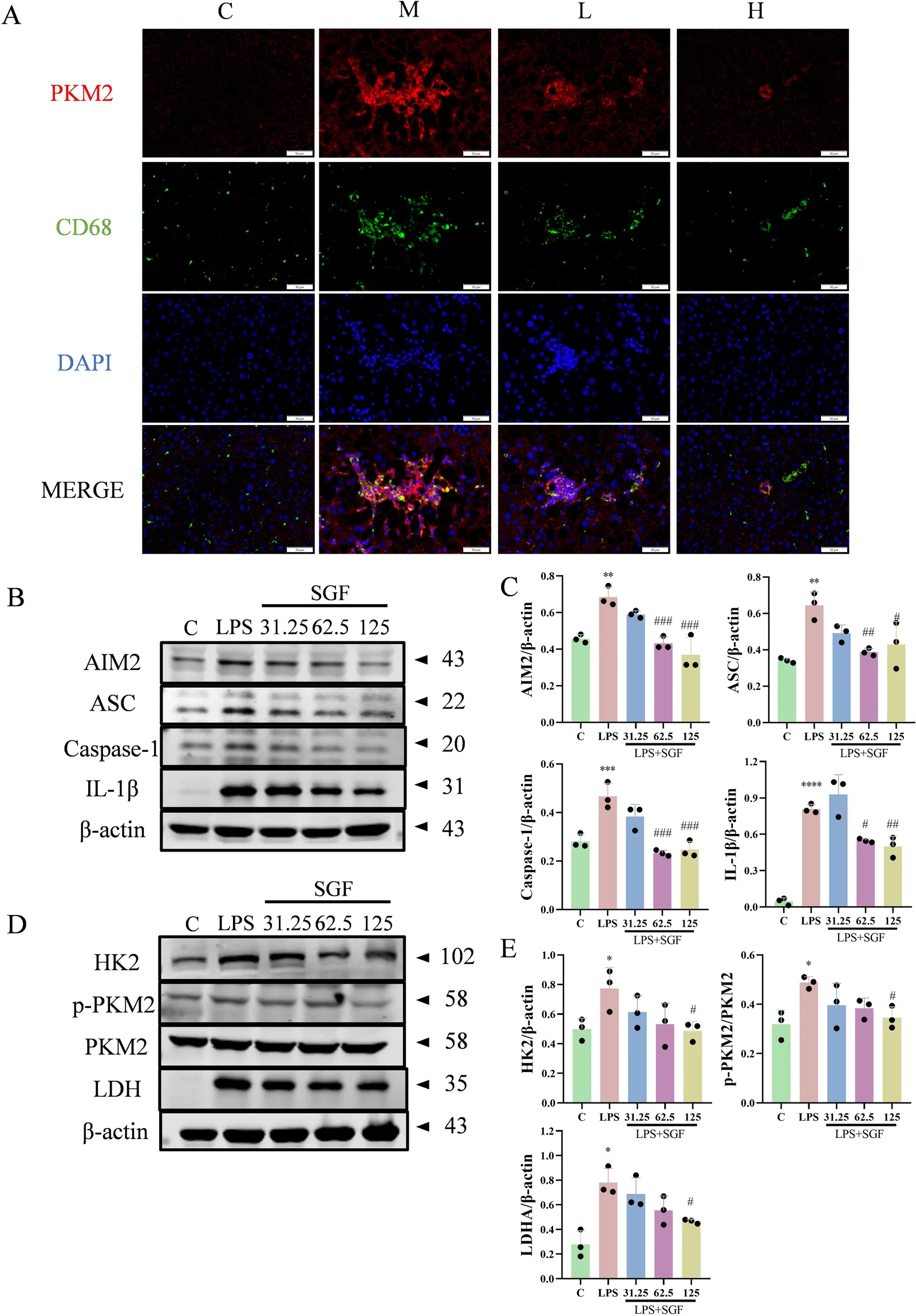
SGF inhibits LPS-induced glycolysis and AIM2 inflammasome activation in RAW264.7 macrophages. **(A)** Co-staining analysis of PKM2 and CD68 on liver tissue. **(B, C)** Protein expression of AIM2, ASC, Caspase-1 and IL-1β. **(D, E)** Protein expression of HK2, p-PKM2, PKM2 and LDHA. Data are presented as the mean ± SD (n = 3). *p < 0.05, **p < 0.01, ***p < 0.001, ****p < 0.0001 vs. C; ^#^*p <* 0.05, ^##^*p* < 0.01, ^###^*p* < 0.001 vs. M.

To confirm the expression of PKM2 in macrophages, we conducted double immunofluorescence staining of PKM2 and the macrophage marker CD68 in murine NASH liver tissues. The results showed that PKM2 expression was significantly higher in macrophages than in hepatocytes, and that SGF treatment effectively downregulated PKM2 expression in hepatic macrophages (**Figure 8A**). To further validate the regulatory effects of SGF on macrophage glycolysis and inflammasome activation, we established an LPS-induced inflammatory model in macrophages for *in vitro* investigation. A time-course experiment was subsequently performed using 1 μg/mL LPS to optimize the modeling time (38). Western blot results showed that AIM2 inflammasome expression reached its peak at 8 h post-LPS stimulation (**Supplementary Figures 1B, C**), while glycolysis-related protein expression increased gradually over time (**Supplementary Figures 1D, E**). Accordingly, 1 μg/mL LPS with an 8 h stimulation time was selected for all subsequent RAW264.7 cell experiments. SGF pretreatment significantly inhibited LPS-induced AIM2 inflammasome activation and glycolysis upregulation in RAW264.7 cells (**Figures 8B–E**). These results confirmed that SGF exerts a prominent anti-inflammatory effect and markedly inhibits LPS-induced upregulation of glycolysis in murine macrophages.

### 3.9 PKM2 overexpression partially abrogates the pharmacological effects of SGF in RAW264.7 macrophages

**Figure 9:**
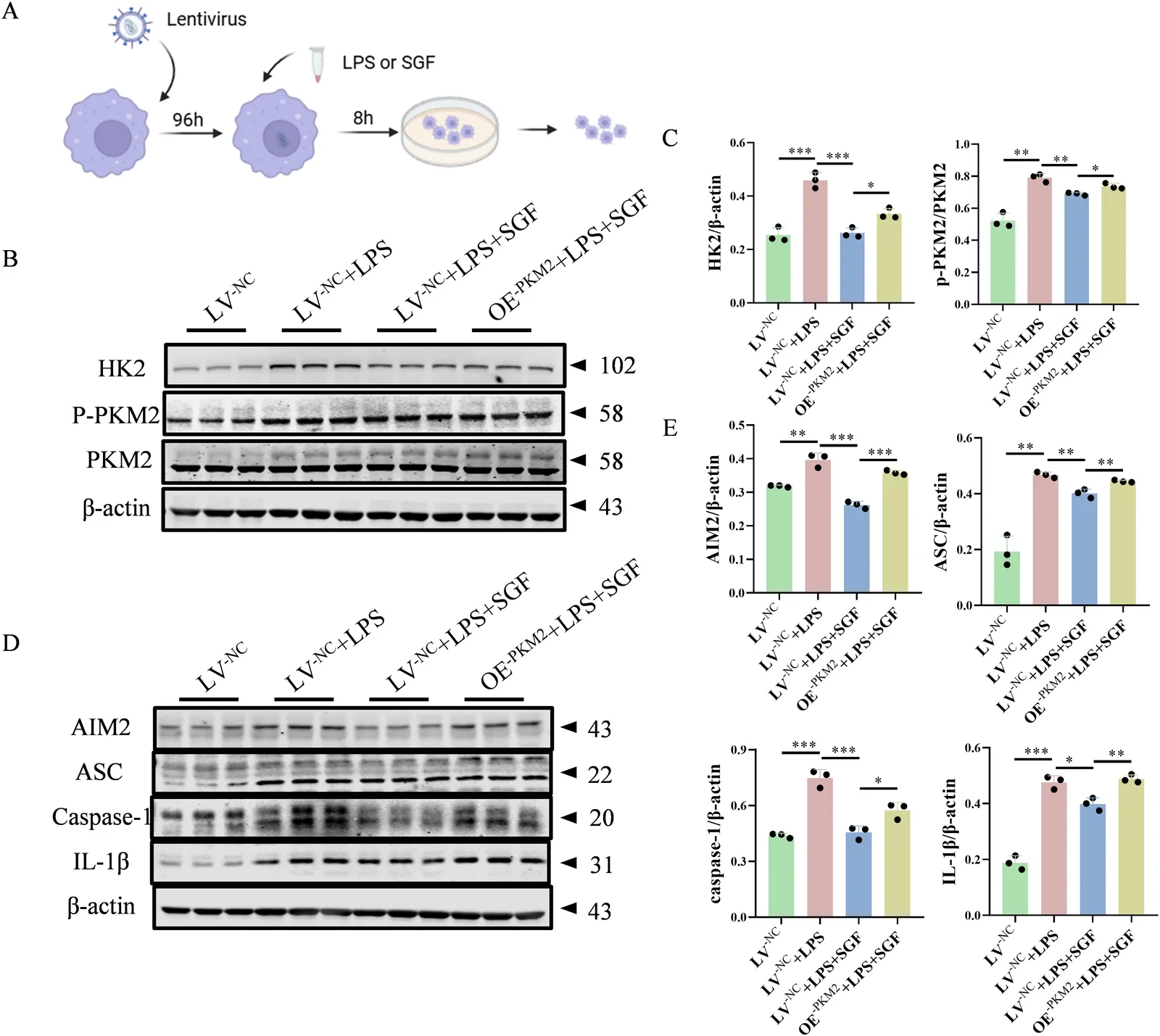
PKM2 overexpression partially abrogates the pharmacological effects of SGF in RAW264.7 macrophages. **(A)** Flowchart depicting the effects of PKM2 overexpression on the pharmacological actions of SGF in RAW264.7 cells. **(B, C)** Protein expression of HK2, p-PKM2, and PKM2. **(D, E)** Protein expression of AIM2, ASC, Caspase-1, and IL-1β. Data are presented as the mean ± SD (n = 3). *p < 0.05, **p < 0.01, ***p < 0.001, ****p < 0.0001.

To further confirm the role of PKM2 in SGF-mediated regulation of the AIM2 inflammasome pathway, we transduced RAW264.7 cells with a PKM2-overexpressing lentivirus (**Figure 9A**) and harvested cells at 8 h post-treatment for protein expression analysis. Western blot analysis showed that PKM2 overexpression partially abrogated SGF’s inhibitory effect on PKM2 phosphorylation (**Figures 9B, C**) and reversed SGF-mediated suppression of AIM2 inflammasome activation (**Figures 9D, E**). In addition, Co-IP results revealed no direct physical interaction between PKM2 and AIM2 (**Supplementary Figure 2D**). Collectively, these findings indicate that PKM2-mediated glycolytic signaling, rather than direct protein-protein binding, drives AIM2 imflammasome activation in macrophages, and SGF suppresses this cascade mainly via inhibiting PKM2 phosphorylation.

### 3.10 Macrophage-derived PKM2 modulates glycolysis and AIM2 inflammasome activation in co-cultured AML-12 hepatocytes

**Figure 10:**
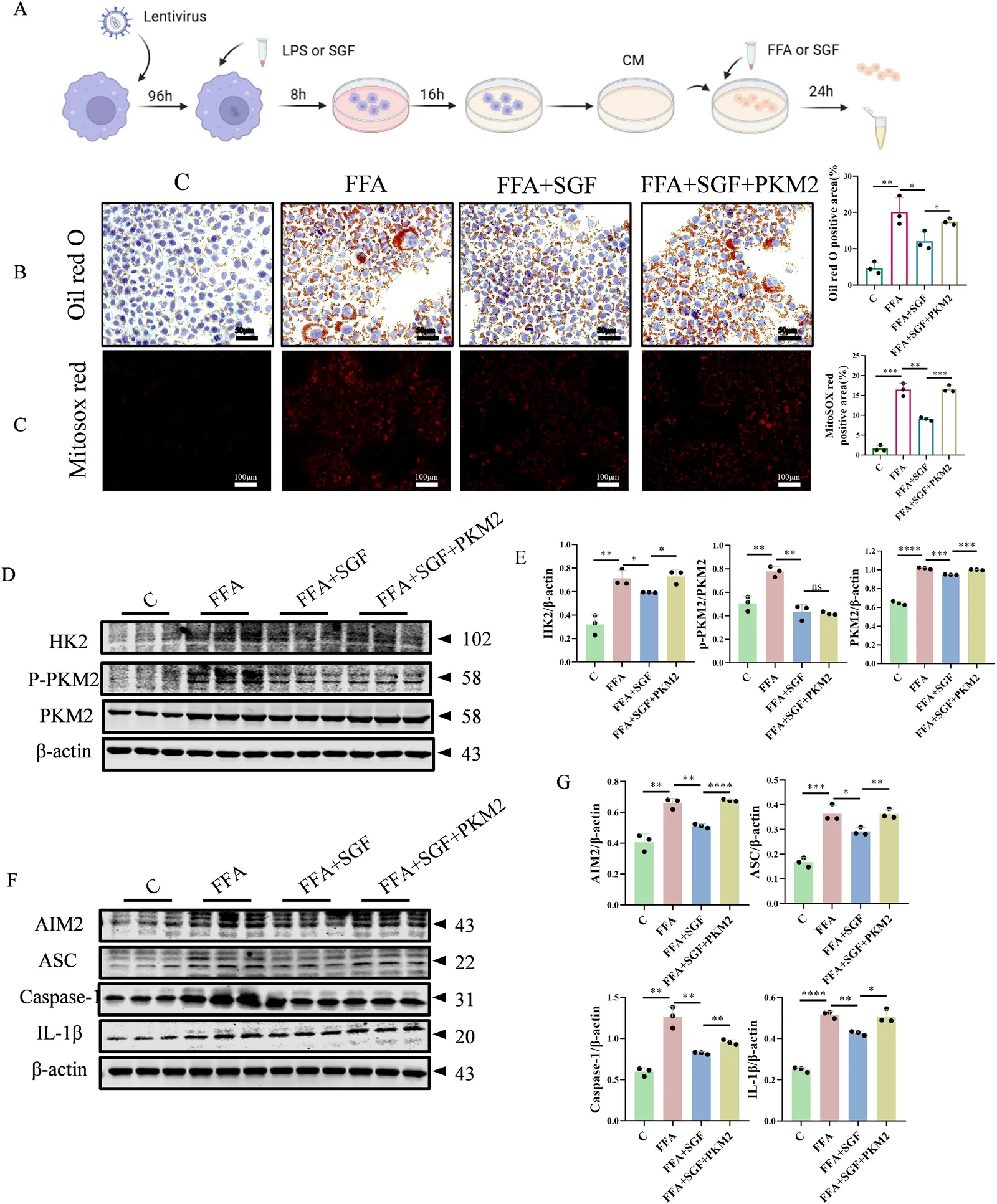
RAW264.7 cells affected glycolysis and inflammasome expression in AML-12 cells. **(A)** Flowchart of the co-culture experiment between RAW264.7 and AML-12 cells. **(B)** Oil Red O staining of AML-12 cells in the co-culture system. **(C)** MitoSOX Red staining of AML-12 cells in the co-culture system. **(D, E)** Protein expression of HK2, p-PKM2, and PKM2 of AML-12 cells in the co-culture system. **(F, G)** Protein expression of AIM2, ASC, Caspase-1, and IL-1β of AML-12 cells in the co-culture system. Data were presented as the mean ± SD (n = 3). *p < 0.05, **p < 0.01, ***p < 0.001, ****p < 0.0001.

To explore whether enhanced glycolysis in macrophages modulates hepatocellular function in the NASH microenvironment, we established a transwell co-culture system of RAW264.7 and AML-12 cells to validate this paracrine interaction (**Figure 10A**). Oil Red O staining showed that PKM2-overexpressing RAW264.7 cells significantly promoted intracellular lipid droplet accumulation in co-cultured AML-12 cells (**Figure 10B**). MitoSOX Red staining also revealed a significant increase in mitochondrial ROS fluorescence intensity in these co-cultured AML-12 cells (**Figure 10C**). Furthermore, PKM2-overexpressing RAW264.7 cells significantly modulated the glycolytic process in co-cultured AML-12 cells. Western blot analysis showed that the protein expression of HK2 and PKM2 was significantly upregulated in AML-12 cells. Notably, this upregulation abrogated the inhibitory effect of SGF on these proteins (**Figures 10D, E**), with no significant effect on PKM2 phosphorylation levels. In addition, the expression of AIM2 inflammasome-related proteins in co-cultured AML-12 cells was significantly altered: AIM2, ASC, caspase-1, and IL-1β protein levels were markedly increased, which reversed the SGF-mediated inhibitory effect on AIM2 inflammasome activation (**Figures 10F, G**). Collectively, alterations in macrophage PKM2 signaling may stimulate the release of paracrine mediators to remodel the hepatic microenvironment, consequently regulating glycolytic reprogramming and AIM2 inflammasome activation in neighboring hepatocytes under NASH conditions.

## 4 Discussion

Non-alcoholic steatohepatitis, an advanced subtype of nonalcoholic fatty liver disease, is pathologically characterized by hepatic steatosis, oxidative stress, chronic inflammation, and metabolic dysregulation. NASH is defined by steatosis, inflammation and hepatocyte injury, while fibrosis represents an optional pathological staging feature rather than an essential diagnostic criterion (39, 40). Although long-term HFD induces milder fibrosis compared with MCD diet models, the 20-week HFD protocol has been well documented to induce progressive early NASH with increased NAS scores and mild collagen deposition in published studies (41, 42). Globally, NASH prevalence continues to rise, yet effective clinical therapeutic options remain limited. Smilax glabra Roxb. is a classic herbal medicine widely applied for metabolic and inflammatory disorders. SGF, its primary bioactive flavonoid components, lower lipids by inhibiting adipocyte lipogenesis and alleviate hepatic lipid accumulation in animal models (30, 43). Nevertheless, the preventive efficacy and detailed mechanisms of SGF against NASH remain poorly defined. In this study, we systematically evaluated SGF protection against HFD-induced NASH in mice and validated molecular targets using hepatocyte and macrophage models *in vitro*. Our data demonstrate that SGF alleviates obesity, glucose-lipid metabolic disturbance and liver injury, accompanied by suppression of excessive hepatic glycolysis and AIM2 inflammasome activation. Mechanistically, SGF exerts hepatoprotective effects via cell-specific inhibition of macrophage PKM2, which further regulates hepatocyte metabolism and inflammation through macrophage–hepatocyte crosstalk. This study reveals a previously unreported cell-dependent regulatory mechanism of SGF and provides experimental evidence supporting SGF as a promising natural agent for NASH intervention.

Disordered hepatic glucose and lipid metabolism initiates steatosis, oxidative stress and inflammatory cascades, which collectively aggravate liver injury and constitute core pathological features of NASH (44). Consistent with previous studies demonstrating the lipid-lowering potential of SGF in rats and zebrafish models (30, 45), our *in vivo* results show that SGF mitigates HFD-induced weight gain and visceral fat accumulation independent of food intake, accompanied by reduced serum glucose, TC, HDL-C and LDL-C. Of note, elevated HDL-C is a recognized paradoxical phenomenon in HFD-fed mice. HFD promotes formation of enlarged, pro-inflammatory HDL particles enriched with acute-phase proteins, facilitating hepatic cholesterol deposition rather than exerting physiological protective effects (46–48). Thus, SGF-mediated HDL-C reduction reflects restored lipid homeostasis. Histological staining (H&E, Oil Red O staining, Masson’s trichrome) and biochemical detection of hydroxyproline, LDH, AST and ALT further confirm that SGF attenuates hepatic steatosis, fibrosis and hepatocyte damage. Oxidative stress links steatosis and inflammation during NASH progression. Our results reveal that SGF increases antioxidant enzyme activity (SOD, CAT) and reduces MDA and intracellular ROS accumulation. Excessive mitochondrial ROS triggers mitochondrial dysfunction, aggravating hepatocyte injury. These observations align with the known antioxidant capacity of SGF and highlight its multi-targeted preventive effects via improving metabolic homeostasis, alleviating oxidative stress and relieving hepatic lesions.

Aberrant glycolysis represents a hallmark of metabolic reprogramming in NASH. Upregulation of glycolytic rate-limiting enzymes correlates closely with steatosis progression and liver injury (14, 49). HK2, PKM2 and LDHA drive glycolytic flux, boosting production of pyruvate and lactate to exacerbate metabolic dysfunction (50–52). Herein, HFD upregulated hepatic HK2, PKM2, LDHA and glycolytic metabolites, whereas SGF effectively blocked abnormal glycolysis. This agrees with studies showing flavonoids modulate glycolysis by targeting key enzymes, indicating SGF as a candidate glycolysis inhibitor for NASH. Lower pyruvate and lactate levels upon SGF treatment also imply improved hepatic metabolite clearance, contributing to reduced metabolite-triggered hepatotoxicity. Persistent inflammation is another central pathological event in NASH, with inflammasomes acting as key mediators of inflammatory responses (53). The NLRP3 inflammasome has been extensively investigated in obesity-related metabolic diseases (54), while the modulation of the AIM2 inflammasome by natural products in the context of NASH remains poorly characterized. SGF exhibits broad anti-inflammatory activity across various disease models (28, 55–57); nevertheless, few studies have explored whether SGF regulates the AIM2 inflammasome during NASH progression. Our data show that HFD feeding upregulated hepatic AIM2, ASC, caspase-1 and IL-1β expression, and SGF treatment markedly blunted AIM2 inflammasome activation. Mechanistically, mitochondrial injury and excessive ROS production drive mtDNA leakage into the cytosol, and cytosolic dsDNA functions as a direct agonist to trigger AIM2 inflammasome assembly. Consistent with this, we observed elevated cytoplasmic mtDNA abundance under FFA exposure, and this alteration was rescued by SGF. By alleviating mitochondrial oxidative stress, SGF limits mtDNA release and consequently restrains excessive AIM2 inflammasome activation. We further validated these findings *in vitro* using LPS-stimulated RAW264.7 macrophages and FFA-challenged AML-12 hepatocytes. Although primary Kupffer cells more faithfully recapitulate the native hepatic immune microenvironment, RAW264.7 macrophages are widely employed in recent immunometabolism studies of NASH owing to their high stability and reproducibility (38, 58–60). LPS is commonly applied to elicit inflammatory responses in macrophages (38, 54, 61), whereas FFA treatment reproduces lipid overload and metabolic stress in hepatocytes, mimicking key features of NASH *in vitro* (62). These observations support the rationality of our cellular experimental systems. As a key rate-limiting enzyme in glycolysis, PKM2 acts as a central regulator linking metabolic reprogramming and inflammatory responses in metabolic diseases, and its high expression in macrophages is closely associated with inflammasome activation (63). Our results uncover cell-specific functions of PKM2 under SGF intervention. In AML-12 hepatocytes, PKM2 knockdown inhibited AIM2 inflammasome activation, yet PKM2 overexpression did not notably reverse SGF’s protective effects. Given the moderate overexpression efficiency achieved in hepatocytes, these data only suggest that PKM2 may serve as a necessary but not sufficient regulator for AIM2 activation in hepatocytes. The phosphorylated active form of PKM2, rather than total PKM2 protein, may dominate AIM2 regulation within hepatocytes. Future studies employing high-efficiency overexpression plasmids or adeno-associated virus-mediated gene delivery are required to rigorously validate the hepatocellular PKM2-AIM2 axis. Immunofluorescence co-localization revealed abundant PKM2 in hepatic macrophages, where SGF significantly inhibited PKM2 expression. Consistently, PKM2 overexpression in RAW264.7 macrophages partially rescued SGF-induced inhibition of glycolysis and AIM2 inflammasome. Transwell co-culture assays further demonstrated that macrophages overexpressing PKM2 promoted lipid deposition, oxidative stress, glycolysis and AIM2 inflammasome activation in co-cultured AML-12 hepatocytes and abolished SGF-mediated hepatoprotection.

These data suggest that macrophage PKM2 initiates paracrine signaling to disrupt hepatocyte homeostasis. PKM2-driven glycolysis promotes the release of pro-inflammatory cytokines (IL-1β, TNF-α) and glycolytic metabolites (lactate, pyruvate), which serve as critical paracrine mediators to exacerbate hepatocyte metabolic dysfunction and injury (64–67). Although the present Transwell system verified macrophage-hepatocyte crosstalk, the exact paracrine factors governing this intercellular regulation remain uncharacterized. Accumulating evidence indicates that macrophage-derived exosomes deliver functional cargoes including miRNAs, proteins and lipids to modulate lipid uptake, glycogen synthesis, fatty acid metabolism and inflammatory responses in hepatocytes, ultimately affecting intracellular lipid droplet accumulation and steatosis progression (68–73). Further investigation focusing on macrophage-derived exosomes will help distinguish exosome-mediated signaling from soluble-factor-mediated effects on hepatocyte lipid metabolism. In addition, Co-IP assays failed to detect direct physical interaction between PKM2 and AIM2, supporting the notion that PKM2 modulates AIM2 inflammasome activation indirectly through glycolytic metabolites rather than protein interaction (66).

Several limitations of this study should be acknowledged. First, the current study adopted a synchronous drug administration design and only evaluated the preventive effects of SGF on early-stage NASH. This concurrent intervention model cannot fully distinguish whether the protective effects of SGF are derived from the improvement of upstream lipid accumulation or specific inhibition of the PKM2-related inflammatory pathway. The therapeutic efficacy against established NASH with severe fibrosis requires further exploration. Second, the PKM2 overexpression efficiency in AML-12 hepatocytes is suboptimal. Although downstream phenotypic changes were observed, the current data can only support correlative alterations rather than rigorous causal conclusions in hepatocytes. Our results imply that PKM2 may act as a necessary but not sufficient regulator for AIM2 inflammasome activation in hepatocytes. Although recent studies using macrophage-specific PKM2 knockout mice have validated the critical role of macrophage PKM2 in NASH progression (74), the present study lacks *in vivo* genetic evidence to consolidate our mechanistic conclusions. Third, this study did not perform pharmacological verification using selective PKM2 inhibitors. Compound 3k, a well-recognized specific PKM2 inhibitor, will be utilized as a superior tool compound in future mechanistic validation. In addition, parallel *in vitro* mechanistic comparison between SGF and empagliflozin was not conducted, which limits the comprehensive evaluation of the relative advantages of SGF and will be supplemented in subsequent work. Fourth, paracrine crosstalk between macrophages and hepatocytes was confirmed by the Transwell co-culture system, yet we cannot distinguish whether the regulatory effects are mediated by soluble cytokines or exosomal cargoes. Further work is required to dissect the contribution of macrophage-derived exosomes to this intercellular signaling. Finally, the precise molecular mechanism by which SGF regulates PKM2 phosphorylation remains unclear, including whether SGF directly interacts with PKM2 or modulates upstream kinases/phosphatases, as well as the exact phosphorylation sites involved. The functional monomeric component of SGF responsible for PKM2–AIM2 axis regulation also requires further identification.

Despite these shortcomings, this study systematically demonstrates for the first time that SGF alleviates HFD-induced NASH by suppressing macrophage PKM2-mediated glycolysis and subsequent AIM2 inflammasome activation through macrophage–hepatocyte communication. We clarify distinct functions of PKM2 in hepatocytes versus macrophages, reveal novel pharmacological mechanisms of SGF, and provide a cell-targeted preventive strategy for NASH.

## 5 Conclusion

In summary, SGF prevents HFD-induced NASH progression by restoring glucose and lipid homeostasis, mitigating oxidative stress and liver damage, and suppressing abnormal glycolysis and AIM2 inflammasome activation. Mechanistically, the hepatoprotective activity mainly relies on cell-specific inhibition of macrophage PKM2, which shapes macrophage–hepatocyte crosstalk within the hepatic microenvironment. These findings highlight SGF as a promising candidate agent for NASH prevention. Meanwhile, macrophage PKM2 is identified as a valuable cellular target, offering new directions for developing cell-type-specific therapeutic strategies against metabolic fatty liver disease.

## Data Availability

The original contributions presented in the study are included in the article/**Supplementary Material**. Further inquiries can be directed to the corresponding author/s.
