# Supplementary material for: Smilax glabra flavonoids ameliorate non-alcoholic steatohepatitis by regulating PKM2-dependent glycolysis and suppressing AIM2 inflammasome activation
Source: Front Immunol. 2026 Sep 9;17:1906439. doi: 10.3389/fimmu.2026.1906439 (PMC13597434; doi:10.3389/fimmu.2026.1906439)
Supplement: Supplementary file 1 [file DataSheet1.docx]

Supplementary Materials

|  | Sequence |
| --- | --- |
| si-NC | Forward：5’-UUCUCCGAACGUGUCACGUTT-3’  Reverse：5’-ACGUGACACGUUCGGAGAATT-3’ |
| si-PKM2 | Forward：5’-CAUGCUGUCUGGAGAAACATT-3’  Reverse：5’-UGUUUCUCCAGACAGCAUDTT-3’ |

**Table S1** siRNA sequences used in this study.

**Table S2** Sequence of the primers for qRT-PCR.

| Gene | Sequence |
| --- | --- |
| β-actin | Forward：5’- CATCCGTAAAGACCTCTATGCCAAC -3’  Reverse：5’- ATGGAGCCACCGATCCACA -3’ |
| IL-1β | Forward：5’- CTCGCAGCAGCACATCAACAAG -3’  Reverse：5’- CCACGGGAAAGACACAGGTAGC -3’ |
| IL-18 | Forward：5’- AAAGTGCCAGTGAACCCCAGAC -3’  Reverse：5’- AGAGAGGGTCACAGCCAGTCC -3’ |
| IL-6 | Forward：5’- ACAAAGCCAGAGTCCTTCAGAGA -3’  Reverse：5’- CTGTTAGGAGAGCATTGGAAATTG -3’ |
| TNF-α | Forward：5’- ATGAGCACAGAAAGCATGATC -3’  Reverse：5’- TACAGGCTTGTCACTCGAATT -3’ |
| Caspase-1 | Forward：5’- CCTGGTCTTGTGACTTGGAGGAC -3’  Reverse：5’- ATCAGCAGTGGGCATCTGTAGC -3’ |
| NLRP3 | Forward：5’- CACCAGCCTCATCAACCAGAAGAAG -3’  Reverse：5’- TCCTCTGCGTTCCTACACTCCTG -3’ |
| HK2 | Forward：5’- CAGTGAACAGGCGGAGAATGGATG -3’  Reverse：5’- GGTGGTGACTGGGAGGGATGG -3’ |
| PKM2 | Forward：5’- TGCCGCCTGGACATTGACTC -3’  Reverse：5’- GACTTAATCATCTCCTTCAGCATCTCC -3’ |
| GLUT1 | Forward：5’- GTGCTCCTCGTGCTCTTCTTCATC -3’  Reverse：5’- CTCCTCGGGTGTCTTGTCACTTTG -3’ |
| LDHA | Forward：5’- CGGCTGGGTCCTGGGAGAAC -3’  Reverse：5’- ACCTCCTTCCACTGCTCCTTGTC -3’ |
| AIM2 | Forward：5’- ACTGCTCTGCTGCCTCTGC -3’  Reverse：5’- GCTTCCTGTTCTGCCACCATC -3’ |
| ASC | Forward：5’- AAGTGGACGGAGTGCTGGATG -3’  Reverse：5’- TGTCTTGGCTGGTGGTCTCTG -3’ |

**Table S3** Antibody information.

| Name | Supplier | Cat No. |
| --- | --- | --- |
| β-actin | proteintech | 81115-1-RR |
| PKM2 | proteintech | 15822-1-AP |
| p-PKM2 | Affinity | DF7772 |
| HK2 | proteintech | 22029-1-AP |
| LDHA | Wanleibio | WL03271 |
| AIM2 | proteintech | 66902-1-Ig |
| AIM2 | ABclonal | A25252 |
| Caspase-1 | proteintech | 22915-1-AP |
| Caspase-1 | Abmart | T510200 |
| ASC | proteintech | 10500-1-AP |
| IL-1β | proteintech | 26048-1-AP |
| NLRP3 | Affinity | DF7438 |
| CD68 | proteintech | 28058-1-AP |

**Figure S1** (A)Cell viability. (B-C) Protein expression of AIM2, ASC and IL-1β. (D-E) Protein expression of HK2, p-PKM2, PKM2 and LDHA. Data were presented as the mean ± SD (n = 3). **p* < 0.05, ***p* < 0.01, ****p* < 0.001, *****p* < 0.0001 vs. C.

**Figure S2** (A)Hepatic hydroxyproline content.(B) Fluorescence staining of dsDNA (green) and Mitotracker (red) in AML-12. (C) Plasmid transfection efficiency and protein validation in AML-12. (D) Co-IP validation. **p* < 0.05, ***p* < 0.01, ****p* < 0.001, *****p* < 0.0001 vs. C, *#p* < 0.05, *##p* < 0.01, *###p* < 0.001, *####p* < 0.0001 vs. M.

## **Supplementary Methods S1. Hydroxyproline assays**

Liver tissues were precisely weighed (0.08–0.1 g) and hydrolyzed with 1 mL of alkaline hydrolysis solution at 95 °C for 20 min according to the manufacturer's instructions (Kit No. A030-2-1, Nanjing Jiancheng Bioengineering Institute, China). After cooling to room temperature, 10 μL of indicator solution was added, followed by 1.0 mL of solution A (pH adjusting reagent I). Solution B (pH adjusting reagent II) was then added dropwise until the solution turned yellow. The mixture was diluted with distilled water to a final volume of 10 mL. An aliquot of 2 mL was transferred and mixed with 30 mg of activated charcoal, then centrifuged at 3500 rpm for 10 min. One milliliter of the supernatant was mixed with 500 μL of reagent I, incubated for 10 min, then mixed with 500 μL of reagent II and incubated for another 10 min. Subsequently, 500 μL of reagent III was added, and the mixture was incubated in a 65 °C water bath for 20 min. After cooling to room temperature, the sample was centrifuged again at 3500 rpm for 10 min. The absorbance of the supernatant was measured at 550 nm. The hydroxyproline content was calculated using the following formula:$Hydroxyproline content(\text{μg/mg)}=\frac{\mathbf{A}\mathrm{sample}-\mathbf{A}\mathrm{blank}}{\mathbf{A}\mathrm{standard}-\mathbf{A}\mathrm{blank}}\times\mathbf{C}standard\times\frac{\mathbf{V}\mathrm{hydrolysate}}{\mathbf{M}\mathrm{liver}}$

## **Supplementary Methods S2. Fluorescence staining of dsDNA and Mitotracker**

The MitoTracker stock solution (C1049B, Beyotime, China) was diluted to the working concentration with serum-free culture medium. After treatment, cells were gently washed once with pre-warmed PBS. The cells were then incubated with the MitoTracker working solution at 37 °C in the dark for 30 min. Following incubation, the staining solution was removed, and the cells were gently washed 2–3 times with pre-warmed serum-free medium to remove unbound dye. Subsequently, the dsDNA dye stock solution (12641ES, Yeasen, China) was diluted to 1:500 with serum-free medium and incubated with the cells at 37 °C in the dark for 30 min. The staining solution was then discarded, and the cells were washed twice. Hoechst dye was then added and incubated for 10 min. After two additional washes with PBS, the stained cells were immediately visualized and photographed using a fluorescence inverted microscope.

## **Supplementary Methods S3. Co-immunoprecipitation (Co-IP) assays**

FFA-induced AML-12 cells were lysed using a commercial Co-IP Kit (Bes3011; Boxin Biotechnology, Guangzhou, China) according to the manufacturer's instructions. The protein-containing supernatant was incubated overnight at 4 °C with gentle agitation on a rotary shaker with one of the following antibodies: anti-PKM2 antibody (1:100), anti-AIM2 antibody (1:100), or non-specific IgG (1:100). This process generated immunoprecipitation complexes for PKM2 and AIM2 pull-downs as an IgG negative control. Protein A/G-MagBeads were then incubated with each protein-antibody complex for 2 h at 4 °C with gentle rotation. After three washes with ice-cold lysis buffer, bound proteins were eluted using Elution buffer (100 °C, 10 min). Western blot analysis was subsequently performed.
